# Prevalence and determinants of obesity among individuals with diabetes in Indonesia

**DOI:** 10.12688/f1000research.125549.2

**Published:** 2023-02-10

**Authors:** Mahalul Azam, Luluk Fadhoh Sakinah, Martha Irene Kartasurya, Arulita Ika Fibriana, Tania Tedjo Minuljo, Syed Mohamed Aljunid

**Affiliations:** 1 Department of Public Health, Faculty of Sports Science, Universitas Negeri Semarang, Semarang, Jawa Tengah, 50229, Indonesia; 2 Department of Public Health Nutrition, Faculty of Public Health, Diponegoro University, Semarang, Jawa Tengah, 50275, Indonesia; 3 Division of Endocrinology and Metabolism, Department of Internal Medicine, Dr. Kariadi General Hospital, Semarang, Jawa Tengah, 50244, Indonesia; 4 Department of Health Policy and Management, Faculty of Public Health, Kuwait University, Kuwait City, 11311, Kuwait

**Keywords:** prevalence, determinant, diabetes mellitus, obesity, Indonesia

## Abstract

**Background:** Obesity and diabetes mellitus (DM), both individually or simultaneously, increase the risk of morbidity and mortality. The present study aimed to determine the prevalence and determinants of obesity among diabetic individuals in Indonesia.

**Methods:** Data were extracted based on 2018 Indonesian Basic Health Survey (Riset Kesehatan Dasar=RISKESDAS). This study involved all individuals with DM and categorized obesity based on body mass index. After data clearing, this study analyzed 3911 DM subjects of the 33.905 subjects acquired from the 2018 RISKESDAS. The study also observed demographic data, diabetes control parameters, history of hypertension, lipid profiles, and food consumption patterns. These variables were involved in a Chi-square test, and related variables were then involved in the Binary logistic regression to define the independent determinants of obesity among DM subjects.

**Results:** Of the 3911 DM subjects included, the study found an obesity prevalence of 32.9%. This study found that female (prevalence odds ratio [POR]=2.15; 95% CI: 1.76-2.62), age 15-44 years (POR=2.46; 95% CI: 1.83-3.33), urban residence (POR=1.49; 95% CI: 1.25-1.77), history of hypertension (POR=1.25; 95% CI: 1.04-1.51), high diastolic blood pressure (POR=1.90; 95% CI: 1.58-2.29), high LDL (POR=1.44; 95% CI: 1.13-1.84), high HDL (POR=0.60; 95% CI: 0.46-0.78, and high triglycerides (POR=1.27; 95% CI: 1.07-1.50) were the risk factor of obesity  among DM subjects; while higher education (POR=0.64; 95% CI: 0.53-0.78) and married (POR=0.73; 95% CI: 0.59-0.90) were protective factors of obesity among DM subjects.

**Conclusions:** The study concluded that almost one-third of DM subjects in Indonesia were obese. Female, age, urban residence, education level, history of hypertension, diastolic blood pressure, and lipid profiles were all associated with obesity among DM subjects in Indonesia. These findings suggest that monitoring and controlling of related determinants is needed to prevent complications caused by the doubled burden of diabetes and obesity.

## Introduction

Non-communicable (NCD) diseases are the most contributed cause of death worldwide. ^1^ Obesity is related to NCD’s increased morbidity and mortality, including diabetes mellitus (DM). ^1^ The global prevalence of obesity in adults has nearly doubled since 1980. ^2^ In 2015, the prevalence of obesity, classified as a body mass index (BMI) ≥ 27 kg/m ^2^ in Indonesia, was 30.4%. ^3^

The prevalence of DM globally was estimated at 9.3% (463 million) in 2019. It is predicted to increase to 10.2% (578 million) in 2030. ^4^ The prevalence of DM in Indonesia in 2014 was 6.7% ^5^ and has increased over the last six years ^6^^,^^7^ placing Indonesia in the 9 ^th^ rank in the world. ^8^ Based on the Indonesian Basic Health Survey (Riset Kesehatan Dasar=RISKESDAS), the DM prevalence rate significantly increased from 6.9% in 2013 to 10.9% in 2018. ^9^ DM accounts for 3% of the causes of death in Indonesia. ^10^

Obesity increases the risk of DM, hypertension, dyslipidemia, stroke, cancer, coronary heart disease, and obstructive sleep apnea. ^2^^,^^11^^–^^13^ Obesity contributes to insulin resistance, which is associated with DM conditions. ^14^ Obesity increased the release of non-esterified fatty acids, glycerol, hormones, and pro-inflammatory cytokines in adipose tissue, affecting insulin resistance conditions. ^15^ Severe obesity in childhood and adolescence is associated with the increased risk of DM in youth and young adults. ^16^ Obesity is related to the high increase in carbohydrate intake that causes insulin resistance in genetically predisposed individuals. ^16^ The obese-year describes the severity and the duration of obesity, and the age of onset was the independent predictor for type 2 DM. ^17^ Overweight and obesity have been associated with the poor control of blood glucose levels, blood pressure, and cholesterol among DM subjects. ^18^ Indeed, obesity is well known as a major modifiable risk factor for type 2 DM. ^11^^,^^19^

A study in Saudi Arabia revealed that the prevalence of obesity among DM was 38.3%, and non-smoker homemakers were the most affected. ^20^ A previous study in Tanzania observed that the majority of type 2 DM patients (85%) were overweight (44.9%) or obese (40.1%). ^21^ Among them, 33.7% were overweight/obese after being diagnosed with type 2 DM, and the prevalence was significantly higher in women. ^21^ In Turkey, the prevalence of obesity among DM individuals in 1999 was 35.6%, which increased with age. ^22^ In the US, the prevalence of obesity among adults with diagnosed DM in 2004 was 54.8%, ^23^ while among young aged less than 20 years old with type 2 DM, most of them were obese (79.4%). ^24^ Previous studies had reported the prevalence of obesity among DM and its related factors ─ which had a pivotal role in the comprehensive management of diabetes subjects as well as describing the risk factors and the poor control of diabetes. However, to our knowledge, no study has reported the prevalence of obesity among DM patients, especially in an extensive national survey in Indonesia. The present study aimed to explore the prevalence and independent determinants of obesity among DM subjects in Indonesia using national survey data.

## Methods

### Study design

Data were extracted from 2018 RISKESDAS, a five-annual national health cross-sectional survey managed by the National Institute of Health Research and Development (NIHRD), Ministry of Health, Republic of Indonesia. RISKESDAS survey was approved by the Ethics Committee of the National Institute of Health Research and Development (NIHRD), Ministry of Health, Republic of Indonesia. At this time, this 2018 survey was the latest RISKESDAS survey conducted. The target sample subjects of RISKESDAS 2018 were the same as the other national surveys conducted by the Indonesian Central Bureau of Statistics. To minimize the selection bias, the sampling was done using the census block system with the target of 300,000 households visited from 30,000 census blocks. The RISKESDAS’ respondents were selected from all household members. The detailed study protocol was published in the official RISKESDAS report ^25^ and other publications. ^26^^,^^27^ In total, respondents in the RISKESDAS were 1,017,290 subjects from 416 districts and 98 cities in 34 provinces.

### Sample and variables

The study population was all individuals with diabetes mellitus, those who were previously diagnosed by a doctor with/without medication, and those who were tested using a rapid plasma glucose test. Fasting plasma glucose ≥ 126 mg/dL or 2 hours postprandial and random plasma glucose levels ≥ 200 mg/dL with classic symptoms of polyuria, polydipsia, polyphagia, and weight loss or blood glucose at 2 hours after a glucose load ≥ 200 mg/dl or the use of glucose-lowering drugs. ^25^ For the subjects aged ≥19 years old, BMI ≥ 27 kg/m ^2^ was categorized as obese, while among the subjects aged 15-18 years old, the z score of BMI for age > 2 were categorized as obese. ^25^ We categorized educational level as high for the subjects who graduated from senior high school or higher and low for those who completed their junior high school or lower. Age was categorized as young adults for subjects aged 15-44 years, middle-aged adults for 45-64 years and senior adults for subjects aged 65 years and over. The detailed food frequency and physical activity questionnaires were published previously in the study protocol. ^25^ The physical activity questionnaire was modified from the WHO Global Physical Activity Questionnaire (GPAQ). The subjects were categorized as “lack of physical activity” if the subjects met the sedentary or low physical activity criteria and “adequate” if the subjects met moderate to vigorous physical activity, which reached ≥ 1500 metabolic equivalent (MET). ^25^ The consumption of sugary food and drinks, salty food, fatty food, burnt food, food with preservatives, food flavoring, carbonated drinks, energy drinks, and instant noodles consumption were categorized as ‘frequent’ if the frequency was 1-2 times a week or more and classified as ‘rare’ if the frequency was less than 1-2 times a week. ^25^

### Data analysis

We performed the chi-square tests, to find the association between each factor and the obesity status. Then, the factors with p-values < 0.1 were included in the multiple logistic regression models, with Enter method, to obtain the independent determinants of obesity among DM subjects. A *p-*value < 0.05 was considered statistically significant. The factors which were associated with obesity, by Chi-square tests with the p-value <0.1, were demographical characteristics (age, sex, occupation, residence status, educational level, marital status), lifestyle factors (physical activity, smoking, medication compliance, fatty food consumption, food flavoring consumption, instant noodles consumption) and biomedical characteristics (fasting plasma glucose level, previous history of hypertension, systolic and diastolic blood pressure, level of total cholesterol, high-density lipoprotein cholesterol, low-density lipoprotein cholesterol, triglycerides). All statistical analyses were performed using the Statistical Package for the Social Sciences (SPSS) software (version 23.0 for Windows, IBM SPSS Inc., Chicago, IL).

The prevalence of DM among RISKESDAS subjects was 8.5% meaning that the number of DM subjects was 86,469 of 1,017,290 RISKESDAS respondents. ^25^ However, after considering the data completeness, consistency, and outliers, this study only analyzed 33,905 total subjects acquired from RISKESDAS 2018 data; of them 3,911 subjects were categorized as having DM (11.5% DM subjects among the total population aged ≥ 15 years acquired data; the original dataset is provided as *Underlying data.* ^68^

## Results

Of the 3,911 DM subjects analyzed in this study, 1,287 (32.9%) subjects were obese. Table 1 shows the characteristics of the study population based on socio-demographic characteristics, i.e., age, sex, occupational status, type of residence, education level, and marital status. Most of the subjects were 45-64 years (57.4%) and 67% were women. Most of the subjects were employed (52.0%), 56.9% had a low education level (<12 education years) and 78.5% were married.

**Table 1.** Subjects’ characteristics.

Characteristics	n=3911	%
**Obesity**		
Yes	1287	32.9
No	2624	67.1
**Age**		
≥65	669	17.1
45-64	2244	57.4
15-44	998	25.5
**Sex**		
Male (total)	1289	33.0
15-44	235	18.2
45-64	756	58.7
≥65	298	23.1
Female (total)	2622	67.0
15-44	763	29.1
45-64	1488	56.8
≥65	371	14.1
**Occupation**		
Unemployed	1878	48.0
Employed	2033	52.0
**Residence**		
Urban	2057	52.6
Rural	1854	47.4
**Education level**		
Low (<12 education years)	2227	56.9
High (≥ 12 education years)	1684	43.1
**Marital status**		
Unmarried	841	21.5
Married	3070	78.5

Factors of obesity among DM subjects are shown in Table 2. This table shows the results of the chi-square tests. We observed subjects’ characteristics, physical activity, smoking status, DM duration, and previous history of hypertension. We found that the age category of 15-44 years had the prevalence odds ratio (POR) of 3.02 with a 95% confidence interval (CI) of 2.39-3.80, and the age category of 45-64 years had a POR of 2.16, with a 95% CI: 1.75-2.68 for obese compared to the age group of ≥ 65 years (as reference), respectively. The proportion of women was significantly higher in the obesity group compared to the non-obesity, with the POR of 2.26; 95% CI: 1.94-2.64. The unemployed group also had a higher risk for obesity with the POR of 1.43 and 95% CI: 1.25-1.64.

**Table 2.** Distribution of demographical characteristics of obesity among DM subjects.

Parameters	Obesity		*p* *	POR (95% CI)
	Yes	No		
**Age**				
15-44 (n;%)	411; 76.5	587; 51.9	**<0.001** **<0.001**	3.02 (2.39-3.80) 2.16 (1.75-2.68)
45-64 (n;%)	750; 85.6	1494; 73.3		
≥65 (n;%) (Ref)	126; 23.5	543; 48.1		
**Sex**				
Female (n;%)	1008; 78.3	1614;61.5	**<0.001**	2.26 (1.94-2.64)
Male (n;%)	279; 21.7	1010; 38.5		
**Occupation**				
Unemployed (n;%)	707; 54.9	1208; 46.0	**<0.001**	1.43 (1.25-1.64)
Employed (n;%)	580; 45.1	1416; 54.0		
**Residence**				
Urban (n;%)	789;61.3	1268; 48.3	**<0.001**	1.69 (1.48-1.94)
Rural (n;%)	498; 38.7	1356; 51.7		
**Education level**				
Low (n;%)	904; 70.2	2102; 80.1	**<0.001**	0.59 (0.50-0.68)
High (n;%)	383; 29.8	522; 19.9		
**Marital status**				
Unmarried (n;%)	216; 16.8	625; 23.8	**<0.001**	0.65 (0.54-0.77)
Married (n;%)	1071; 83.2	1999; 76.2		
**Physical activity**				
Low (n;%)	1118; 86.9	2188; 83.4	**<0.001**	1.31 (1.09-1.60)
Adequate (n;%)	169; 13.1	436; 16.6		
**Smoking**				
Yes (n;%)	220; 17.1	781; 29.8	**<0.001**	0.49 (0.41-0.58)
No (n;%)	1067; 82.9	1843; 70.2		
**DM duration**				
<5 years (n;%)	601; 46.7	1280; 48.8	0.23	0.92 (0.81-1.05)
≥5 years (n;%)	686; 53.3	1344; 51.2		
**Previous history of hypertension**				
Yes (n;%)	351; 34.2	491; 26.3	**<0.001**	1.46 (1.24-1.72)
No (n;%)	675; 65.8	1379; 73.7		

* Chi square test.

Table 3 showed that high fasting plasma glucose (POR=1.21, 95% CI: 1.06-1.39), high systolic blood pressure (POR=1.49, 95% CI: 1.30-1.70), high diastolic blood pressure (POR=2.46, 95% CI: 2.15-2.82), high total cholesterol level (POR=1.44, 95% CI: 1.26-1.64), high low-density lipoprotein (LDL) cholesterol level (POR=1.83, 95% CI: 1.50-2.24), and high triglycerides level (POR=1.72, 95% CI: 1.50-1.97) were related to the obesity among DM subjects as risk factors with POR>1, while high high-density lipoprotein (HDL) cholesterol level (POR=0.58, 95% CI: 0.47-0.73) was related as a protective factor. The study also reported that the result of the chi-square test for the parameters of dietary patterns and expressed that frequent fatty food consumption (POR=1.26, 95% CI: 1.09-1.45), food flavoring consumption (POR=1.23, 95% CI: 1.02-1.49), and instant noodle consumption (POR=0.81, 95% CI: 0.68-0.96) were correlated to the obesity among DM subjects ( Table 4).

**Table 3.** Distribution of diabetes mellitus control parameters of obesity among DM subjects.

Parameters	Obesity		*p* *	POR (95% CI)
	Yes	No		
**Medication compliance**				
No (n;%)	39;14.7	64; 10.6	0.09	0.69 (0.45-1.06)
Yes (n;%)	226; 85.3	538; 89.4		
**Fasting plasma glucose**				
≥126 mg/dL	647; 52.7	1192; 47.9	**0.01**	1.21 (1.06-1.39)
<126 mg/dL	580; 47.3	1295; 52.1		
**2 hours postprandial plasma glucose**				
<200 mg/dL	901; 76.0	1884;77.7	0.26	0.91 (0.77-1.07)
≥200 mg/dL	284; 24.0	540; 22.3		
**Systolic blood pressure**				
≥140 mmHg	736; 57.3	1242; 47.4	**<0.001**	1.49 (1.30-1.70)
<140 mmHg	549; 42.7	1377; 52.6		
**Diastolic blood pressure**				
≥90 mmHg	743; 57.8	947; 35.8	**<0.001**	2.46 (2.15-2.82)
<90 mmHg	542; 42.2	1682; 64.2		
**Total cholesterol**				
≥200 mg/dL	681; 52.9	1151; 43.9	**<0.001**	1.44 (1.26-1.64)
<200 mg/dL	606; 47.1	1473; 56.1		
**HDL cholesterol**				
≥60 mg/dL	118; 9.2	388; 14.8	**<0.001**	0.58 (0.47-0.73)
<60 mg/dL	1169; 90.8	2236; 85.2		
**LDL cholesterol**				
≥100 mg/dL	1148; 89.2	2148; 81.9	**<0.001**	1.83 (1.50-2.24)
<100 mg/dL	139; 10.8	476; 18.1		
**TG cholesterol**				
≥150 mg/dL	645; 50.1	967; 36.9	**<0.001**	1.72 (1.50-1.97)
<150 mg/dL	642; 49.9	1657; 63.1		

* Chi square test; HDL: high-density lipoprotein; LDL: low density lipoprotein cholesterol; TG: triglyserides.

**Table 4.** Distribution of dietary patterns of obesity among DM subjects.

Dietary patterns	Obesity		*p* *	PR (95% CI)
	Yes	No		
**Sugary food consumption**				
Frequent (n;%)	625; 48.6	1302; 49.6	0.53	0.96 (0.84-1.10)
Rare (n;%)	662; 51.4	1322; 50.4		
**Sugary drinks consumption**				
Frequent (n;%)	816; 63.4	1688;64.3	0.57	0.96 (0.84-1.10)
Rare (n;%)	471; 36.6	936; 35.7		
**Salty food consumption**				
Frequent (n;%)	607; 47.2	1227; 46.8	0.42	1.06 (0.89-1.16)
Rare (n;%)	680; 52.8	1397; 53.2		
**Fatty food consumption**				
Frequent (n;%)	886; 68.8	1673; 63.8	**0.001**	1.26 (1.09-1.45)
Rare (n;%)	401; 31.2	951; 36.2		
**Burnt food consumption**				
Frequent (n;%)	116; 9.0	269; 9.8	0.22	0.87 (0.69-1.09)
Rare (n;%)	1171; 91.0	2355; 89.7		
**Food with preservatives consumption**				
Frequent (n;%)	121; 9.4	225; 8.6	0.39	1.11 (0.88-1.39)
Rare (n;%)	1166; 90.6	2399; 91.4		
**Food flavoring consumption**				
Frequent (n;%)	1110; 86.2	2194;83.6	**0.03**	1.23 (1.02-1.49)
Rare (n;%)	177; 13.8	430; 16.4		
**Carbonated drinks consumption**				
Frequent (n;%)	48; 3.7	85; 3.2	0.43	1.16 (0.81-1.66)
Rare (n;%)	1239; 96.3	2539; 96.8		
**Energy drinks consumption**				
Frequent (n;%)	31; 2.4	63; 2.4	0.99	1.00 (0.65-1.55)
Rare (n;%)	1256; 97.6	2561; 97.6		
**Instant noodles consumption**				
Frequent (n;%)	218; 16.9	529; 20.2	**0.02**	0.81 (0.68-0.96)
Rare (n;%)	1069; 83.1	2095; 79.8		
**Alcohol**				
Yes (n;%)	12; 0.9	25; 1	0.95	0.98 (0.49-1.95)
No (n;%)	1275; 99.1	2599; 99		

* Chi-square test.

The final model of the binary logistic regression is shown at Table 5. Of the 20 variables that had been analyzed, ten parameters had statistically significant POR. Female sex (adjusted (a)POR=2.15; 95% CI: 1.76-2.62), urban residence (aPOR=1.49; 95% CI: 1.25-1.77), higher education level (aPOR=0.64; 95% CI: 0.53-0.78), married (aPOR=0.73; 95% CI: 0.59-0.90), history of hypertension (aPOR=1.25; 95% CI: 1.04-1.51), high diastolic blood pressure (aPOR=1.90; 95% CI: 1.58-2.29), high triglycerides level (aPOR=1.27; 95% CI: 1.07-1.50), high LDL cholesterol level (aPOR=1.44; 95% CI: 1.13-1.84), high HDL cholesterol level (aPOR=0.60; 95% CI: 0.46-0.78), and younger age (aPOR=0.63; 95% CI: 0.54-0.73) altogether were related to the obesity among DM subjects (R=0.39).

**Table 5.** Binary logistic regression of determinants for obesity among DM subjects.

Determinants	Adjusted POR	*P* *	95% CI	
Female	2.15	<0.001	1.76	2.62
Age 15-44 years	2.46	<0.001	1.83	3.33
Age 45-64	1.53	<0.001	1.19	1.96
Age ≥ 65 years (ref)	NA	NA	NA	NA
Urban residence	1.49	<0.001	1.25	1.77
High education level	0.64	<0.001	0.53	0.78
Married	0.73	0.004	0.59	0.90
History of hypertension	1.25	0.020	1.04	1.51
High SBP ≥ 140 mmHg	1.19	0.088	0.97	1.46
High DBP ≥ 90 mmHg	1.90	<0.001	1.58	2.29
High LDL cholesterol	1.44	0.004	1.13	1.84
High HDL cholesterol	0.60	<0.001	0.46	0.78
High triglyceride	1.27	0.005	1.07	1.50
Fatty food consumption	1.13	0.17	0.95	1.34

DBP: diastolic blood pressure; HDL: high-density lipoprotein; LDL: low density lipoprotein cholesterol; POR: prevalence Odds Ratio; SBP: systolic blood pressure.

R ^2^=0.39 (Nagelkerke).

* Binary logistic regression test.

## Discussion

Our cross-sectional study revealed that the prevalence of obesity among DM subjects in Indonesia was 32.9%. The current study had a national scope, using community-based data representing the figure of obesity among DM subjects in Indonesia. A study based on diabetes clinics data in Tanzania reported that the prevalence of obesity among DM subjects was 40.1%, and the prevalence of overweight was 44.9%. ^21^ A study based on diabetes clinic data in Ghana revealed that the prevalence of overweight and obesity among DM subjects was 32%. ^28^ The prevalence of obesity among DM subjects is higher than in the general adult population. The prevalence in the general adult population in Indonesia (2015), Tanzania (2020), and Ghana (2017) were: 30.4%, ^3^ 37.8%, ^29^ and 29.9%, ^30^ respectively. Previous studies have concluded that obesity is associated with the incidence of diabetes mellitus and strongly correlates with the duration and onset of obesity. ^17^^,^^31^^,^^32^ The difference in the prevalence of obesity among DM subjects and the general population in the three countries is consistent. Unfortunately, the data is not supported by prevalence data according to the onset and duration of obesity. We also recognized that our study did not address the incidence of pregnancy in reproductive-age women, which posed a risk of bias. The incidence of pregnancy among reproductive-age women in Indonesia was 7.48%, ^9^ while the population of reproductive-age women was 1153 or 29.48% among the DM population in our dataset. Assuming the incidence of pregnancy of reproductive-age women is 7.48%, we ignored around 86 of our female respondents who may be pregnant.

This study found that female diabetes subjects were more likely to be obese than males, with an adjusted-POR of 2.15; 95% CI: 1.76-2.62. This relationship aligns with the study conducted in the diabetic clinic in Tanzania, ^21^ which concluded that women are five times more likely to be obese than men. ^21^ A cohort study in the US ^33^ also indicated that women were more at risk of obesity in diabetes than men and tended to have more medical expenditure. The prevalence of obesity continues to increase yearly, and women have increased faster than men. ^34^ A previous study in a rural population in India ^35^ also revealed that the prevalence of overweight and obesity was higher in women than in men. This study ^35^ also concluded that abdominal and central obesity was more prevalent in women with diabetes subjects. A study in Thailand ^36^ observed diabetes subjects with chronic kidney disease (CKD), and disclosed that the prevalence of obesity in this entire study population was 51.5% (68.2% in women and 31.8% in men, *p*=0.01). This study also concluded that the prevalence of obesity was lower in CKD than in non-CKD diabetes subjects (46.5% *vs.* 54.1%, *p*<0.001), and there was no difference based on the stages of CKD. ^36^ Previous studies have shown consistent results that the prevalence of obesity in women with diabetes is higher than in men; this might be influenced by the pre-menopause/menopause status as the dominant study population being observed (56.8%), i.e., 45-64 years of age ( Table 1), of which the median age of menopause in Indonesia is 51 years. ^37^^–^^39^ Decreasing estrogen levels at menopause causes shifting the gynoid to android fat, which makes fat distribution dominant to abdominal fat; furthermore, the basal metabolic rate will also be very low. ^37^

The current study concluded that age category 45-64 years significantly had a higher risk to get obese among DM subjects (a-POR=1.53; 95% CI: 1.19-1.96) than the age category ≥ 65 years, so did the age category 15-44 years which had an almost twofold odds ratio (a-POR=2.46; 95% CI: 1.83-3.33). Previous studies reported that prevalence of overweight and obesity was highest in the age category 41-60 years, ^21^^,^^40^ The previous studies coincided with the current study finding that revealed a high prevalence of obesity was observed in the age category ≤ 60 years. However, the current study found that the highest prevalence was in the 15-44 years group, while previous studies found the highest prevalence was in the 41-60 years group. ^21^^,^^40^ Age category on young and middle adults had a high risk to get obese among DM subjects. The younger age groups tend to be obese in this study; it can be associated with an increased prevalence of obesity in children ^41^ and might be related to the onset and duration of diabetes mellitus. ^17^^,^^31^^,^^32^

This study also found that living in urban residences was more likely to get obese among diabetic subjects. The urban area is a complex area where environmental, social, cultural, and economic factors significantly impact the health of the area’s population—both positive and negative impacts on the health sector. ^42^ While living in urban areas has a positive role in education and the economy, living in an urban area often causes greater risk factors that threaten health damage due to greater exposure to all sectors. ^42^ Our study found that living in urban areas has 1.49 times (95% CI: 1.25-.177) the risk of obesity in diabetes subjects than living in rural areas. This study is in accordance with the study in Tanzania ^21^ that deduced diabetes subjects who live in urban areas were 1.3 times more likely to have obesity than diabetes subjects who live in rural areas. The study in Peru ^43^ revealed similar findings in the general population. The subjects living in urban areas were more likely to get obese; more detail, this study also found that longer residing in urban areas for the rural-to-urban migrants tended to be obese.

The relationship between education and obesity in the general population and diabetes subjects consistently concluded that lower education tends to get obese. ^44^^–^^46^ Our study also found that lower education levels in diabetes subjects tend to be obese. High-level education related to the understanding and healthier lifestyle that influence obesity status. ^45^ A cross-sectional study in Saudi Arabia conducted the subgroup analysis and concluded that the highest risk of obesity was in the population with high income and low education levels. ^46^ Another study ^47^ observed that the Chinese twins concluded that education positively correlated with obesity, marital status, age, and sex confounders. Higher education will relate to knowledgeable persons. Furthermore, this knowledge and lifestyle are closely related to the incidence of obesity among DM subjects. ^48^ Poor knowledge will affect the poor lifestyle, ^48^ i.e., taking more snacks outside of regular meals, eating late at night, physical inactivity, excessive fast food intake, and alcoholic beverage intake, which were associated with increased obesity among DM subjects. ^48^

It is known as common knowledge that obesity has been firmly associated with profound health consequences and metabolic disorders, including hypertension, diabetes, cardiovascular diseases and dyslipidemia. ^21^^,^^49^ Hypertension was reported as a common co-morbidity of diabetes affecting around 20-60 % of diabetes subjects and twice as prevalence in diabetes subjects. ^28^ Our study found that previous history of hypertension was more likely to get obesity among DM subjects than subjects without a history of hypertension with the a-POR=1.25; 95% CI: 1.04-1.51. The prevalence of hypertension among diabetes was two-three-fold higher than in non-diabetes subjects. ^22^ Similarly, the prevalence of obesity among diabetes was also increasing. The increasing prevalence of obesity is followed by a rising prevalence of type 2 diabetes and other compounds’ health risk. ^50^ These conditions reflect the poor lifestyle of the general population. ^50^

**Figure 1.**
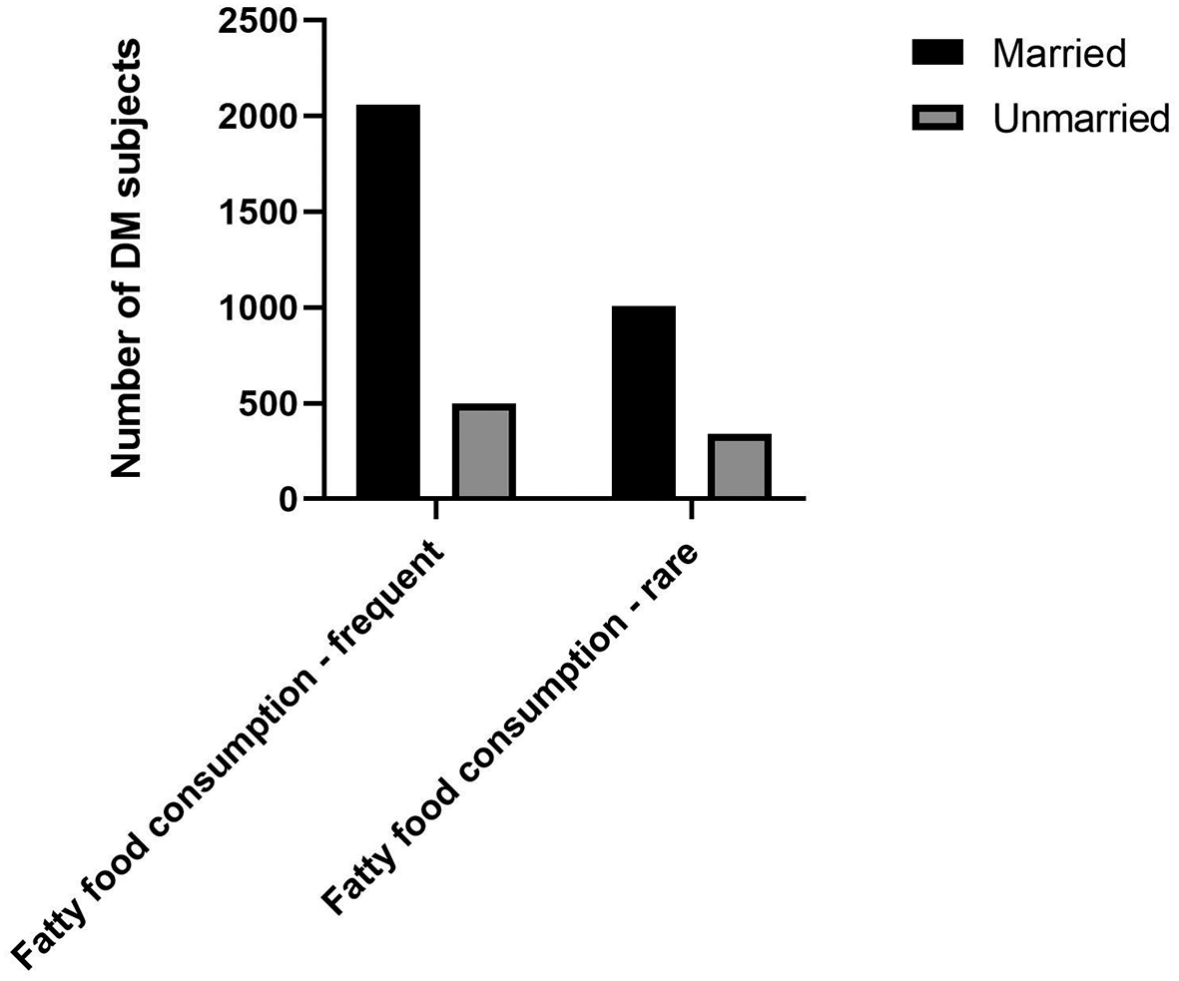
Proportion of fatty food consumption among marital status.

We also noticed that DM subjects that were unmarried significantly had lower odds risk of being obese; a-POR=0.73; 95% CI: 0.59-0.90. This finding was in accordance with the study involving the general population in Malaysia ─ which concluded that never married individuals had a higher risk of being obese. ^51^ However, our finding contradicted a previous study, which concluded that individuals with married status had a higher risk of being obese in the general population. ^52^ In the general population of ever-married women in Bangladesh, a study reported that obesity among them was influenced by oldest age, higher wealth and higher education. ^53^ Similarly, a study in Kuwait that included the general population found that obesity is related to smoking, hypertension, higher income, being women, greater age, and being married. ^54^ A study in the US involving Asian subjects revealed that married or living with a partner was associated with obesity among general populations. ^55^ Marital status describes a general condition that is difficult to be associated with the incidence of obesity; marital status should be described and detailed of their marital quality. A study in the US disclosed that low marital quality was related to diabetes and other health problems. ^56^ Our study also found that a higher proportion of DM subjects with married status had the habit of frequent fatty foods consumption ( Figure 1).

**Figure 2.**
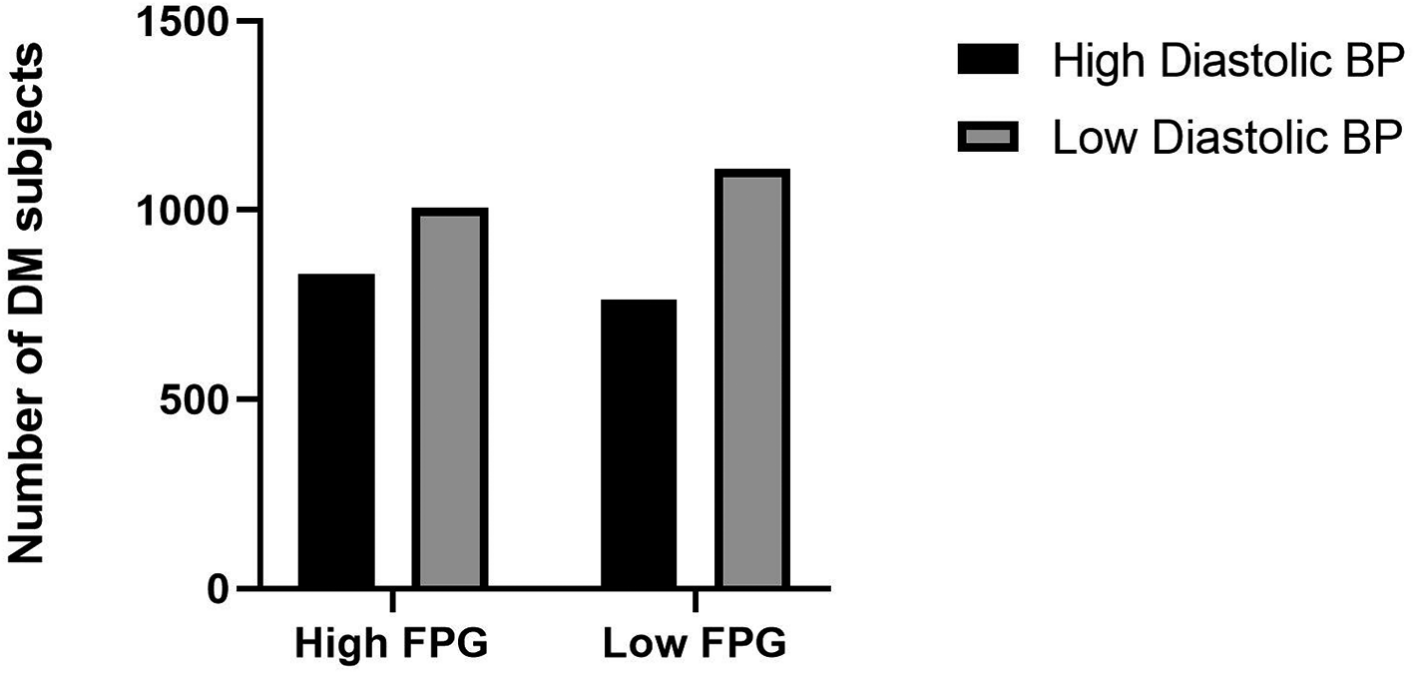
Proportion of fasting plasma glucose levels and diastolic blood pressure. BP: blood pressure, FPG: fasting plasma glucose.

It is firmly known that obesity in DM subjects is associated with poor control of blood glucose levels, blood pressure, and cholesterol. ^48^^,^^57^ The current study found that high diastolic blood pressure (a-POR=1.90; 95% CI: 1.58-2.29), high LDL cholesterol (a-POR=1.44; 95% CI: 1.13-1.84), high HDL cholesterol (a-POR=0.60; 95% CI: 0.46-0.78), and high triglycerides (a-POR=1.27; 95% CI: 1.07-1.50) were related to the occurrence of obesity among DM subjects in this setting. High diastolic blood pressure, high LDL cholesterol, and high triglycerides increase the risk of obesity, while high HDL cholesterol decreases the risk. Obesity itself was the most important modifiable risk factor for DM; obesity also interferes with the treatment and control of dyslipidemia, hyperglycemia, hypertension, and cardiovascular diseases. ^28^ Obesity is also intimately associated with dyslipidemia and hypertension. ^58^^,^^59^ The current study consistently revealed that lipid profiles included LDL cholesterol, HDL cholesterol, and triglycerides related to obesity among DM subjects. Total cholesterol was also associated with obesity among DM in the chi-Square test; however, this association was not statistically significant in the binary logistic regression. Unfortunately, novel lipid biomarkers in obesity, such as proprotein convertase subtilisin/kexin type 9 (PCSK9) and other biomarkers of obesity-associated dyslipidemia were not evaluated in the RISKESDAS 2018 survey. ^58^ Pathophysiology of dyslipidemia observed in obesity was also well known and involved multifactorial parameters, including hepatic overproduction of very low-density lipoprotein, decreased circulating triglycerides lipolysis, and impaired peripheral free fatty acids trapping. ^60^ Adequate exercise and reduce saturated fatty acids intake will be the first choice for treatment of dyslipidemia and simultaneously with obesity. However, medical therapy can be considered if these lifestyle changes could not be sufficiently undertaken. The current study showed the high diastolic blood pressure category has a high proportion of fasting plasma glucose levels ( Figure 2).

Plasma glucose parameters with obesity simultaneously represent the control treatment results for DM subjects. ^58^ However, our study reported that both fasting and 2 hours of postprandial plasma glucose were not related to obesity among DM subjects. Fasting plasma glucose in the chi-square was associated with obesity (POR=1.21; 95% CI: 1.06-1.39) ( Table 3). However, it was not involved in the final binary logistic regression model. Previous studies reported that glycaemic control was proportionate with the body weight and body composition in the diabetes population. ^61^ Another study reported that a lower glycaemic index diet was related to reducing glycaemic control parameters, i.e., glycated hemoglobin (HbA1C), fasting, and postprandial plasma glucose, which was in line with lowering body weight. ^62^ The high postprandial hyperglycemia is usually strongly influenced by carbohydrate intake. This might be why obesity is common in DM subjects with postprandial hyperglycemia. ^62^ Two parameters of fasting and postprandial plasma glucose level were the standard parameters of glycaemic control. Unfortunately, this study did not provide glycated hemoglobin data, which will elucidate this relationship.

Regarding the diet pattern, our study revealed that frequent fatty food consumption was related to obesity in the chi-square test with a POR of 1.26; 95% CI: 1.09– 1.45). We did not find any relationship between obesity in diabetic individuals with sugary food and drink, salty food, food with preservatives and flavoring, instant noodles, carbonated drinks, energy drinks, and alcohol consumption. A cohort study in the general population concluded a relationship between fatty and sugary food consumption and obesity. ^63^ Another cross-sectional study observed the children and adolescents population in Korea revealed that dietary sugars from milk and fruit were inversely related to obesity, while sugar-sweetened beverages increase the risk of obesity in the boy population. ^64^ High salt intake was also concluded as an independent risk factor of obesity in the general population, ^65^ while there is no evidence in our study population. Soft drink consumption was concluded significantly with global overweight, obesity, and diabetes in the general population; ^65^ however, our diabetes population in this study did not find any relationship. Instant noodle consumption in the general population was also reported to be related to the increased risk of obesity and cardiovascular diseases; ^66^^,^^67^ however, as our finding, there was no relationship in our diabetes individuals population.

Our cross-sectional study was conducted to analyze secondary data from RISKESDAS 2018 survey. The availability of parameters in RISKESDAS 2018 data limits our discussion to elucidate comprehensive determinants of obesity in diabetes individuals. Some diabetes therapy that may be used, like insulin and sulphonylurea, is weight gain, so further sub-analysis is also needed on whether this population includes DM subjects administered these drugs when data is provided in the following survey. RISKESDAS 2018 data determined diabetes status just on the determination of subjects’ statement of being previously diagnosed by a doctor or being determined from the measurement of fasting or postprandial plasma glucose, without differing type 1 or type 2 DM.

## Conclusions

The prevalence of obesity among diabetic individuals based on Indonesian RISKESDAS 2018 data was 32.9%. Female sex, age categorized as 15-44 years, living in an urban residence, low education level, previous history of hypertension, high diastolic blood pressure, high LDL and triglycerides level, and low HDL cholesterol level all together were associated with obesity among diabetes individuals in Indonesia. These findings suggest that monitoring and controlling of related determinants is needed to prevent complications caused by the doubled burden of diabetes and obesity.

## Data availability

Figshare: Prevalence and Determinants of Obesity among Indonesian Diabetics. https://doi.org/10.6084/m9.figshare.20291934. ^68^

Data are available under the terms of the Creative Commons Attribution 4.0 International license (CC-BY 4.0).
