# Supplementary material for: Prevalence and determinants of obesity among individuals with diabetes in Indonesia
Source: F1000Res. 2023 Oct 16;11:1063. Originally published 2022 Sep 16. [Version 4] doi: 10.12688/f1000research.125549.4 (PMC10624955; doi:10.12688/f1000research.125549.4)
Supplement: Peer review history

# Reviewer report: Reviewer response for version 4

**DOI:** 10.5256/f1000research.157413.r215706

**Published:** 2023-11-03

**Authors:** Emmanuel Ekpor

**Affiliations:** 1 University of Ghana, Accra, Greater Accra Region, Ghana

Thank you for revising the manuscript as recommended. The paper looks good, except for some sentence structures which need to be improved. Kindly proofread your work and correct any grammatical or sentence errors.

Is the work clearly and accurately presented and does it cite the current literature?

Partly

If applicable, is the statistical analysis and its interpretation appropriate?

Yes

Are all the source data underlying the results available to ensure full reproducibility?

Yes

Is the study design appropriate and is the work technically sound?

Yes

Are the conclusions drawn adequately supported by the results?

Partly

Are sufficient details of methods and analysis provided to allow replication by others?

Yes

Reviewer Expertise:

Diabetes

I confirm that I have read this submission and believe that I have an appropriate level of expertise to confirm that it is of an acceptable scientific standard.

# Reviewer report: Reviewer response for version 3

**DOI:** 10.5256/f1000research.150600.r208478

**Published:** 2023-09-27

**Authors:** Emmanuel Ekpor

**Affiliations:** 1 University of Ghana, Accra, Greater Accra Region, Ghana

This study provides an in-depth analysis of the prevalence and determinants of obesity among individuals with diabetes in Indonesia. However, there are some areas that warrant further consideration and exploration.

 Introduction

- In the second paragraph, the sentence “The prevalence of cause of death by DM in Indonesia in 2014 was 6.7% ^5^ and has increased over the last six years ^6, 7^ placing Indonesia in the 9 ^th^ rank in the world ^8^” lacks clarity. Consider restructuring it as “In 2014, diabetes mellitus (DM) was responsible for 6.7% of all recorded deaths in Indonesia. Over the subsequent six years, this percentage increased, leading to Indonesia's rank rising to 9th in the world for DM-related mortality.”
  Within the same paragraph, these two statements “The prevalence of cause of death by DM in Indonesia in 2014 was 6.7%” and “DM accounts for 3% of the causes of death in Indonesia” are confusing because they seem contradictory in terms of the percentages mentioned. Clarify the differences.
- While you mention that no study has reported the prevalence of obesity among DM patients in Indonesia, you could emphasize the research gap and the significance of your study more explicitly. Why is this information important, and what potential impact could it have on healthcare or policy?

Method

- Reference the WHO Global Physical Activity Questionnaire (GPAQ) in your methods section to provide readers with a clear source for the questionnaire used.
- Create a flowchart to illustrate the participant selection process described in the second paragraph under "data analysis." This visual aid will help readers better understand the methodology.

Discussion

- Clarify whether the studies you are comparing your obesity prevalence with also used the obesity threshold of "BMI ≥ 27 kg/m2," and discuss any differences in thresholds or methodologies. This will provide context for your findings.
- Explore existing systematic reviews or meta-analyses related to your study topics and discuss how your research contributes to or aligns with their findings. If no such papers exist, underscore the need for future research in this area and provide a link to a relevant paper ( https://doi.org/10.1080/07853890.2023.2182909) that supports your argument.
- Specify whether the data on obesity from the RISKESDAS dataset was measured or self-reported by participants. Differentiate between measured and self-reported data, highlighting the potential implications for data accuracy and limitations.

General

- Break down long sentences into shorter, more digestible forms to enhance readability and facilitate comprehension.
- Use transition sentences to connect ideas and paragraphs, improving the overall flow of your paper and helping readers navigate through your work.
- Carefully review your text for grammar and punctuation errors to ensure professionalism and clarity in your writing.

Is the work clearly and accurately presented and does it cite the current literature?

Partly

If applicable, is the statistical analysis and its interpretation appropriate?

Yes

Are all the source data underlying the results available to ensure full reproducibility?

Yes

Is the study design appropriate and is the work technically sound?

Yes

Are the conclusions drawn adequately supported by the results?

Partly

Are sufficient details of methods and analysis provided to allow replication by others?

Yes

Reviewer Expertise:

Diabetes

I confirm that I have read this submission and believe that I have an appropriate level of expertise to confirm that it is of an acceptable scientific standard, however I have significant reservations, as outlined above.

## References

[1] : Prevalence and associated factors of overweight and obesity among persons with type 2 diabetes in Africa: a systematic review and meta-analysis. *Annals of Medicine* .2023;55(1) : 10.1080/07853890.2023.2182909 696-713 10.1080/07853890.2023.2182909 PMC997025136821504

# Response

**Published:** 2023-10-11

**Authors:** Mahalul Azam

*This study provides an in-depth analysis of the prevalence and determinants of obesity among individuals with diabetes in Indonesia. However, there are some areas that warrant further consideration and exploration.*

 Thank you for providing us reviews so that we can improve the quality of our work; We tried to fulfill the reviews and provided the point-by-point revision as follows.

*General*

- *Break down long sentences into shorter, more digestible forms to enhance readability and facilitate comprehension.*
- Thank you for the input, we revised as the advise. The revised statement is provided paralel with the previous revision
- *Use transition sentences to connect ideas and paragraphs, improving the overall flow of your paper and helping readers navigate through your work.*
- Thank you for the input, we revised as the advise. The revised statement is provided paralel with the previous revision.
- *Carefully review your text for grammar and punctuation errors to ensure professionalism and clarity in your writing*.
- Thank you for the input, we revised as the advice. The revised statement is provided paralel with the previous revision.

Introduction

- *In the second paragraph, the sentence “The prevalence of cause of death by DM in Indonesia in 2014 was 6.7%* *5* *and has increased over the last six years* *6* *,* *7* *placing Indonesia in the 9th rank in the world* *8* *” lacks clarity. Consider restructuring it as “In 2014, diabetes mellitus (DM) was responsible for 6.7% of all recorded deaths in Indonesia. Over the subsequent six years, this percentage increased, leading to Indonesia's rank rising to 9th in the world for DM-related mortality.”*
- This sentence revised into “In 2014, diabetes mellitus (DM) was responsible for 6.7% of all recorded deaths in Indonesia. Over the subsequent six years, this percentage increased, leading to Indonesia's rank rising to 9th in the world for DM-related mortality”
*Within the same paragraph, these two statements “The prevalence of cause of death by DM in Indonesia in 2014 was 6.7%” and “DM accounts for 3% of the causes of death in Indonesia” are confusing because they seem contradictory in terms of the percentages mentioned. Clarify the differences.*

We deleted the statement “DM accounts for 3% of the causes of death in Indonesia. ^10^^ “ ^ because this fact is based on the data in 2010; the information already represented by the above statement

  

- *While you mention that no study has reported the prevalence of obesity among DM patients in Indonesia, you could emphasize the research gap and the significance of your study more explicitly. Why is this information important, and what potential impact could it have on healthcare or policy?*
- We explain this importance in the previous sentences, such as:
  - Obesity is related to NCD’s increased morbidity and mortality, including diabetes mellitus (DM).
  - Obesity increases the risk of DM
  - Obesity contributes to insulin resistance, which is associated with DM conditions. ^14^ Obesity increased the release of non-esterified fatty acids, glycerol, hormones, and pro-inflammatory cytokines in adipose tissue, affecting insulin resistance conditions. ^15^ Severe obesity in childhood and adolescence is associated with the increased risk of DM in youth and young adults. ^16^ Obesity is related to the high increase in carbohydrate intake that causes insulin resistance in genetically predisposed individuals. ^16^ The obese-year describes the severity and the duration of obesity, and the age of onset was the independent predictor for type 2 DM. ^17^ Overweight and obesity have been associated with the poor control of blood glucose levels, blood pressure, and cholesterol among DM subjects. ^18^ Indeed, obesity is well known as a major modifiable risk factor for type 2 DM. ^11^^ ,^^19^
  - Previous studies had reported the prevalence of obesity among DM and its related factors ─ which had a pivotal role in the comprehensive management of diabetes subjects as well as describing the risk factors and the poor control of diabetes.

Method

- *Reference the WHO Global Physical Activity Questionnaire (GPAQ) in your methods section to provide readers with a clear source for the questionnaire used.*

We have added the WHO Global Physical Activity Questionnaire (GPAQ) as reference.

  

- *Create a flowchart to illustrate the participant selection process described in the second paragraph under "data analysis." This visual aid will help readers better understand the methodology.*

We had added the subject recruitment in Figure 1; and we made changes of figure sequence, respectively.

 Discussion

- *Clarify whether the studies you are comparing your obesity prevalence with also used the obesity threshold of "BMI ≥ 27 kg/m2," and discuss any differences in thresholds or methodologies. This will provide context for your findings*.
- We add the statement to provide the infromation regarding the determination of obese as BMI ≥ 27 kg/m ^2^ (in the RISKESDAS and Indonesian studies) compare to the other studies which is use the cut off BMI ≥ 30  kg/m ^2^
- All the revised statement are as follows:
  Our cross-sectional study revealed that the prevalence of obesity among DM subjects in Indonesia was 32.9%. The current study had a national scope, using community-based data representing the figure of obesity among DM subjects in Indonesia. A study based on diabetes clinics data in Tanzania reported that the prevalence of obesity (categorized for BMI ≥ 30 kg/m ^2^) among DM subjects was 40.1%, and the prevalence of overweight (categorized for BMI=25-29.9 kg/m ^2^) was 44.9%. ^21^ A study based on diabetes clinic data in Ghana revealed that the prevalence of overweight (categorized for BMI=25-29.9 kg/m ^2^) and obesity (categorized for BMI ≥ 30 kg/m ^2^) among DM subjects was 32%. ^28^ The prevalence of obesity among DM subjects is higher than in the general adult population. The prevalence in the general adult population in Indonesia (2015), Tanzania (2020), and Ghana (2017) were: 30.4%, ^3^ 37.8%, ^29^ and 29.9%, ^30^ respectively. The difference in the prevalence of obesity among DM subjects in the general population in the three countries is consistent, although this RISKESDAS study ^25^ and Indonesian study ^3^ categorized BMI ≥27 kg/m ^2^ as obese. Previous studies have also concluded that obesity is associated with the incidence of diabetes mellitus and strongly correlates with the duration and onset of obesity. ^17^^ ,^^31^^ ,^^32^^  ^Unfortunately, the data is not supported by prevalence data according to the onset and duration of obesity. We also recognized that our study did not address the incidence of pregnancy in reproductive-age women, which posed a risk of bias. The incidence of pregnancy among reproductive-age women in Indonesia was 7.48%, ^9^ while the population of reproductive-age women was 1,153 or 29.48% among the DM population in our dataset. Assuming the incidence of pregnancy of reproductive-age women is 7.48%, we ignored around 86 of our female respondents who may be pregnant.
- *Explore existing systematic reviews or meta-analyses related to your study topics and discuss how your research contributes to or aligns with their findings. If no such papers exist, underscore the need for future research in this area and provide a link to a relevant paper (* *https://doi.org/10.1080/07853890.2023.2182909* *) that supports your argument.*
- We add this statement and add Ekpor E, Akyirem S, Adade Duodu P. Prevalence and associated factors of overweight and obesity among persons with type 2 diabetes in Africa: a systematic review and meta-analysis. Ann Med. 2023 Dec;55(1):696–713 to provide that our work adds information regarding this finding, although little is known regarding systematic review and meta-analysis on this topic in Indonesia.
- *Specify whether the data on obesity from the RISKESDAS dataset was measured or self-reported by participants. Differentiate between measured and self-reported data, highlighting the potential implications for data accuracy and limitations*.
- We add the statement “ The measurement of weight and height were performed previously to define BMI.” to clarify that the BMI was defined by measurement, not self-reported results.

Thank you once again for the review and we hope our revisions can fulfill the review's inputs and improve the quality of our work.

# Reviewer report: Reviewer response for version 3

**DOI:** 10.5256/f1000research.150600.r177211

**Published:** 2023-08-17

**Authors:** Laurentia K. Mihardja

**Affiliations:** 1 National Research and Innovation Agency, Jakarta, Indonesia; 2 Medical Faculty, Malahayati University, Bandar Lampung, Indonesia

Dear Author,

 Thanks for the revisions that have been made. The revisions are good, except for the bottom of the analysis data, namely:

 This study only analyzed 33,905 total subjects acquired from RISKESDAS 2018 data; of them, 3,911 subjects were categorized as having DM (11.5% DM subjects among the total population aged ≥ 15 years acquired data); the original dataset is provided as Underlying data. The sentence about 11.5% DM subjects among the total population aged ≥ 15 years acquired data; should be deleted so that the readers do not misunderstand, because we know that the national prevalence in Riskesdas data is not only calculated based on proportion, but also includes the value of the representative coefficient of samples that have been successfully examined from each province.

 Thank you for your attention

 Best regards,

 Laurentia

Is the work clearly and accurately presented and does it cite the current literature?

Yes

If applicable, is the statistical analysis and its interpretation appropriate?

Partly

Are all the source data underlying the results available to ensure full reproducibility?

Yes

Is the study design appropriate and is the work technically sound?

Yes

Are the conclusions drawn adequately supported by the results?

Yes

Are sufficient details of methods and analysis provided to allow replication by others?

Yes

Reviewer Expertise:

Public Health about Non Communicable Diseases

I confirm that I have read this submission and believe that I have an appropriate level of expertise to confirm that it is of an acceptable scientific standard.

# Reviewer report: Reviewer response for version 2

**DOI:** 10.5256/f1000research.144113.r162988

**Published:** 2023-05-26

**Authors:** Laurentia K. Mihardja

**Affiliations:** 1 National Research and Innovation Agency, Jakarta, Indonesia; 2 Medical Faculty, Malahayati University, Bandar Lampung, Indonesia

Thank you for your attention.

 You have revised according to the suggestions but I still found some statements that I just realized were wrong. I hope you are happy to revise a few things e.g.:

**Abstract, Results**: " *married (POR=0.73; 95% CI: 0.59 -0.90)*", change to "unmarried (POR=0.73; 95% CI: 0.59 -0.90)". Unmarried had lower odds risk of being obese.

**Introduction**: " *The prevalence of DM in Indonesia in 2014 was 6.7%*", change to "The prevalence of cause of death by DM in Indonesia in 2014 was 6.7%" (based on the data from the Sample Registration System, 2014).

**Methods**: add "The number of pregnant women was ignored." Note: it should be accurate if pregnant women should be excluded. Considering that pregnant women of childbearing age (15-49 years) are only 3.6% ( BKKBN 2021, 2018 data was not found), while the sample includes all women and men aged 15 years and over, so pregnant women can be ignored.

**Data Analysis**: " *The prevalence of DM among RISKESDAS subjects was 8.5% meaning that the number of DM subjects was 86,469 of 1,017,290 RISKESDAS respondents*" - This calculation must be corrected: The prevalence of DM was 10.9% in the population aged 15 years and over. The 1,017,290 RISKESDAS samples include residents aged 1 year and over (all samples). Blood sampling was taken only in 2500 Census Blocks (24,980 households) as a subsample for people aged 15 years and over. Please calculate again. (Riskesdas 2018 report, page 9) ^1^

**Discussion**: " *We also noticed that DM subjects that were unmarried significantly had lower odds risk of being obese; a-POR=0.73; 95% CI: 0.59-0.90. This finding was in accordance with the study involving the general population in Malaysia*" - This sentence must be changed to: "This finding was **different **from the study involving the general population in Malaysia."

Is the work clearly and accurately presented and does it cite the current literature?

Yes

If applicable, is the statistical analysis and its interpretation appropriate?

Partly

Are all the source data underlying the results available to ensure full reproducibility?

Yes

Is the study design appropriate and is the work technically sound?

Yes

Are the conclusions drawn adequately supported by the results?

Yes

Are sufficient details of methods and analysis provided to allow replication by others?

Yes

Reviewer Expertise:

Public Health about Non Communicable Diseases

I confirm that I have read this submission and believe that I have an appropriate level of expertise to confirm that it is of an acceptable scientific standard, however I have significant reservations, as outlined above.

## References

[1] : National Report on Basic Health Research, 2018. *Jakarta: NIHRD* .2019;

# Response

**Published:** 2023-06-04

**Authors:** Mahalul Azam

Thank you very much for a very constructive advanced reviews.

 We try the best to address all reviews as follows

 You have revised according to the suggestions but I still found some statements that I just realized were wrong. I hope you are happy to revise a few things e.g.:

**Abstract, Results**: " *married (POR=0.73; 95% CI: 0.59 -0.90)*", change to "unmarried (POR=0.73; 95% CI: 0.59 -0.90)". Unmarried had lower odds risk of being obese.

*We revised the manuscript as suggested*

**Introduction**: " *The prevalence of DM in Indonesia in 2014 was 6.7%*", change to "The prevalence of cause of death by DM in Indonesia in 2014 was 6.7%" (based on the data from the Sample Registration System, 2014).

*We revised the manuscript as suggested*

**Methods**: add "The number of pregnant women was ignored." Note: it should be accurate if pregnant women should be excluded. Considering that pregnant women of childbearing age (15-49 years) are only 3.6% ( BKKBN 2021, 2018 data was not found), while the sample includes all women and men aged 15 years and over, so pregnant women can be ignored.

*We add the information in the manuscript as suggested*

**Data Analysis**: " *The prevalence of DM among RISKESDAS subjects was 8.5% meaning that the number of DM subjects was 86,469 of 1,017,290 RISKESDAS respondents*" - This calculation must be corrected: The prevalence of DM was 10.9% in the population aged 15 years and over. The 1,017,290 RISKESDAS samples include residents aged 1 year and over (all samples). Blood sampling was taken only in 2500 Census Blocks (24,980 households) as a subsample for people aged 15 years and over. Please calculate again. (Riskesdas 2018 report, page 9) ^1^

*We revised the calculation as suggested and revised as follows*

 The prevalence of DM among RISKESDAS subjects aged >15 years old based on rapid plasma glucose test was 10.9% meaning that the number of DM subjects was 4,083 of 37,460 RISKESDAS respondents whose was tested. ^25^ After considering the data completeness, consistency, and outliers, this study only analyzed 33,905 total subjects acquired from RISKESDAS 2018 data; of them 3,911 subjects were categorized as having DM (11.5% DM subjects among the total population aged ≥ 15 years acquired data; the original dataset is provided as *Underlying data.* ^68^

**Discussion**: " *We also noticed that DM subjects that were unmarried significantly had lower odds risk of being obese; a-POR=0.73; 95% CI: 0.59-0.90. This finding was in accordance with the study involving the general population in Malaysia*" - This sentence must be changed to: "This finding was **different **from the study involving the general population in Malaysia."

*We revised the manuscript as suggested*

# Reviewer report: Reviewer response for version 1

**DOI:** 10.5256/f1000research.137868.r150740

**Published:** 2022-12-14

**Authors:** Laurentia K. Mihardja

**Affiliations:** 1 National Research and Innovation Agency, Jakarta, Indonesia; 2 Medical Faculty, Malahayati University, Bandar Lampung, Indonesia

This article is good but needs revision.

1. Based on the Indonesian Basic Health Survey (Riset Kesehatan Dasar= RISKESDAS), the DM prevalence rate significantly increased from 6.9% in 2013 to **10.9%** in 2018 (as in the Riskesdas report which states 10.9% based on ADA 2015 and PERKENI 2015) ^1^.
2. Diabetes Mellitus was defined in Riskesdas 2018 (Basic Health Research in Indonesia 2018) as fasting blood glucose level ≥ 126 mg/dL or random blood glucose ≥ 200 mg/dL with classic symptoms of polyuria, polydipsia, polyphagia, and weight loss or blood glucose at 2 hours after a glucose load ≥ 200 mg/dl or the use of glucose-lowering drugs. If only random blood glucose > 200 mg/dl without classic symptoms, it is not stated as diabetes. I'm sure the authors knew about this statement.
3. All individuals with DM, not those who are pregnant. There must be a certainty that the analyzed individuals have DM without pregnancy. Chances of getting pregnant can be biased. This data must be excluded. The data on obesity needs to be checked again.
4. *"DM subjects that were married significantly had lower odds risk of being obese" - *DM subjects that were **unmarried** significantly had lower odds risk of being obese. Obese in unmarried group 216/ (216 + 625) = 25.6% (Table 2) and obese in married group 1071/ (1071+ 1999) = 34.8%. Continue chi-square and log multivariate regression, we will find unmarried had lower OR than married.  So 7 ^th^ paragraph in the discussion should be revised.
5. Inside Table 5 is written urban residence 1.49 but, underneath the table in the description, is written 1.87.

Thank you for your attention.

**A little summary and opinion**

 The prevalence of Diabetes Mellitus in Indonesia has been rising more rapidly, e.g. the prevalence of Diabetes Mellitus in 2007 among those aged 15 years and over was 5.7% in urban, rising to 10.9% in urban-rural by 2018. Obesity is one of the most potent risk factors for type 2 diabetes. Research showed that the prevalence of overweight and obesity (BMI > 25) in Indonesia among those at the age of more than 18 years was, based on Riskesdas data, 28.9% in 2013 and 35.4% in 2018. The study aimed to determine the prevalence of obesity among diabetes patients. Some studies have been carried out, but there is no national data in Indonesia yet. The result shows a high prevalence of obesity was found in diabetic patients (32.9%) (make sure it doesn't include pregnant individuals). Diabesity will accelerate the occurrence of complications such as heart disease. The high prevalence of diabesity is a warning for program makers that they should increase public awareness to prevent obesity and diabetes.

Is the work clearly and accurately presented and does it cite the current literature?

Yes

If applicable, is the statistical analysis and its interpretation appropriate?

Partly

Are all the source data underlying the results available to ensure full reproducibility?

Yes

Is the study design appropriate and is the work technically sound?

Yes

Are the conclusions drawn adequately supported by the results?

Yes

Are sufficient details of methods and analysis provided to allow replication by others?

Yes

Reviewer Expertise:

Public Health about Non-Communicable Diseases

I confirm that I have read this submission and believe that I have an appropriate level of expertise to confirm that it is of an acceptable scientific standard, however I have significant reservations, as outlined above.

## References

[1] : National Report on Basic Health Research, 2018. *Jakarta: NIHRD* .2019;

# Response

**Published:** 2023-02-06

**Authors:** Mahalul Azam

Thank you very much for the very precise and constructive reviews of our manuscript.

 We have revised it in line with the reviewer's comments as follows:

  

- *Based on the Indonesian Basic Health Survey (Riset Kesehatan Dasar= RISKESDAS), the DM prevalence rate significantly increased from 6.9% in 2013 to **10.9%** in 2018 (as in the Riskesdas report which states 10.9% based on ADA 2015 and PERKENI 2015) ^1^ .*

Thank you for the precise correction, we revised the prevalence rate in 2018 to 10.9%.

  

- *Diabetes Mellitus was defined in Riskesdas 2018 (Basic Health Research in Indonesia 2018) as fasting blood glucose level ≥ 126 mg/dL or random blood glucose ≥ 200 mg/dL with classic symptoms of polyuria, polydipsia, polyphagia, and weight loss or blood glucose at 2 hours after a glucose load ≥ 200 mg/dl or the use of glucose-lowering drugs. If only random blood glucose > 200 mg/dl without classic symptoms, it is not stated as diabetes. I'm sure the authors knew about this statement.*

Thank you very much for the thorough correction; we revised the statement with the additional clause: with classic symptoms of polyuria, polydipsia, polyphagia, and weight loss or blood glucose at 2 hours after a glucose load ≥ 200 mg/dl or the use of glucose-lowering drugs.

  

- *All individuals with DM, not those who are pregnant. There must be a certainty that the analyzed individuals have DM without pregnancy. Chances of getting pregnant can be biased. This data must be excluded. The data on obesity needs to be checked again.*

Thank you. We added the statement in the discussion section: “We recognize that our study did not address the incidence of pregnancy in reproductive-age women, which posed a risk of bias. The incidence of pregnancy among reproductive-age women in Indonesia was 7.48%, while the population of reproductive-age women was 1153 or 29.48% among the DM population in our dataset. Assuming the incidence of pregnancy of reproductive-age women is 7.48%, we ignored around 86 of our female respondents who may be pregnant."

  

- *"DM subjects that were married significantly had lower odds risk of being obese" ****- ****DM subjects that were **unmarried** significantly had lower odds risk of being obese. Obese in unmarried group 216/ (216 + 625) = 25.6% (Table 2) and obese in married group 1071/ (1071+ 1999) = 34.8%. Continue chi-square and log multivariate regression, we will find unmarried had lower OR than married.  So 7 ^th^ paragraph in the discussion should be revised.*

Thank you very much for the correction. We revised as: “We also noticed that DM subjects that were unmarried significantly had lower odds risk of being obese; a-POR=0.73; 95% CI: 0.59-0.90”

- *Inside Table 5 is written urban residence 1.49 but, underneath the table in the description, is written 1.87.*

Thank you very much for the correction. We revised as stated on Table 5: “urban residence (aPOR=1.49; 95% CI: 1.25-1.77)”
